# fMRI Beyond the Clinic: Will It Ever Be Ready for Prime Time?

**DOI:** 10.1371/journal.pbio.0020150

**Published:** 2004-06-15

**Authors:** Richard Robinson

## Abstract

Functional magnetic resonance imaging offers the promise of peeking into the human mind. What signals in the human brain can we really detect and how should the technology be used?

Functional magnetic resonance imaging—fMRI—opens a window onto the brain at work. By tracking changes in cerebral blood flow as a subject performs a mental task, fMRI shows which brain regions “light up” when making a movement, thinking of a loved one, or telling a lie. Its ability to reveal function, not merely structure, distinguishes fMRI from static neuroimaging techniques such as CT scanning, and its capacity to highlight the neural substrates of decisions, emotions, and deceptions has propelled fMRI into the popular consciousness. Discussions of the future of fMRI have conjured visions of mind-reading devices used everywhere from the front door at the airport terminal to the back room of the corporate personnel office. At least one “neuromarketing” research firm is already trying to use fMRI to probe what consumers “really” think about their clients' products.

But will fMRI's utility in the real world ever match the power we currently imagine for it? Is fMRI likely to leave the clinic for widespread use in the courtroom or the boardroom? Are there neuroethical nightmares just around the corner? Or are all these vivid specters really just idle speculations that will never come to pass?

## 150,000 Grains of Rice

To understand the potential, and the limitations, of fMRI, it's helpful to know how the technique works. The heart of the apparatus is a large donut-shaped magnet that senses changes in the electromagnetic field of any material placed in its center, in particular—when a head is scanned—the blood as it flows through the brain. When a region of the brain is activated, it receives an increased flow of oxygenated blood (the extremely rapid redirection of blood within the active brain is one of the underappreciated wonders supporting neural activity). This influx of oxygenated blood alters the strength of the local magnetic field in proportion to the increase in flow, which is detected and recorded by the imaging machinery.

**Figure 1:**
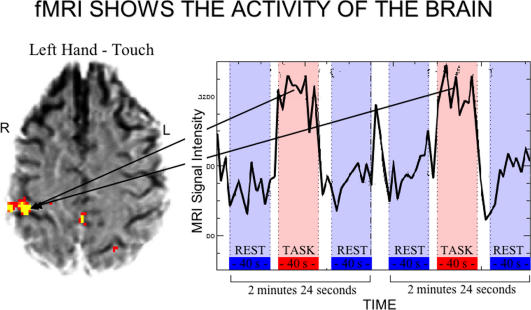
The Basics of fMRI Blood oxygen level–dependent signals are measured and compared between test and resting conditions. (Image courtesy of Joy Hirsch, Columbia University.)

The resolution of the best fMRI machines—the smallest “volume picture element,” or voxel, they can distinguish and make an image of—is currently about 1.5 mm ×1.5 mm × 4 mm, the size of a grain of rice. There are approximately 150,000 of these little volumes in the typical brain, and the immense computers hooked up to the scanners record and integrate signals from all of them. In a typical experiment, a subject, lying still with his head surrounded by the magnet, does nothing for thirty seconds, then performs some task for thirty seconds, then lies still for thirty seconds. For each voxel, the signal during the task is compared to the signal at rest; those areas of the brain with stronger signals during the task are presumed to be processing the information that underlies the performance of the task (Figure 1). According to Joy Hirsch, Director of the Functional Magnetic Resonance Imaging Research Center at Columbia University, fMRI represents a “quantum leap” over any previous technology for imaging the brain. “It enables us for the first time to probe the workings of a normal human brain,” she says. “It's really opening the black box.”

The first caveat about fMRI's imaging power, though, and one that every neuroimager stresses, is that a voxel is a long way from a neuron. There are an estimated 100 billion neurons, so at best, an fMRI is signaling blood flow changes associated with the increased activity of tens of thousands of neurons. As a result, says Hirsch, fMRI “falls short when we want to ask about more detailed brain processes. We're not learning that much about how neurons are doing local computing.” While resolution will improve over time, it seems unlikely that fMRI will ever detect the activity of individual neurons, and so its ability to dissect the “fine structure” of thought is inherently limited. (Even should it become possible to detect and integrate the workings of every neuron in the brain, it would still be far from clear how neuronal firing patterns translate into coherent, perceived thoughts, and this gap is unlikely to be bridged by any advance in imaging technology alone.)

These limitations have not prevented fMRI researchers from making some major discoveries about brain function, however. Hirsch, for instance, showed in one study that minimally conscious individuals still process human speech, and in another, that those who become bilingual as young children employ overlapping language areas in the cerebral cortex, while those who learn a second language later in life use a different area for the second language. The key strength of fMRI, she says, is that it provides the ability to test these kinds of hypotheses about structure–function relationships in the normal brain.

## All Sizes Do Not Fit One

**Figure 2:**
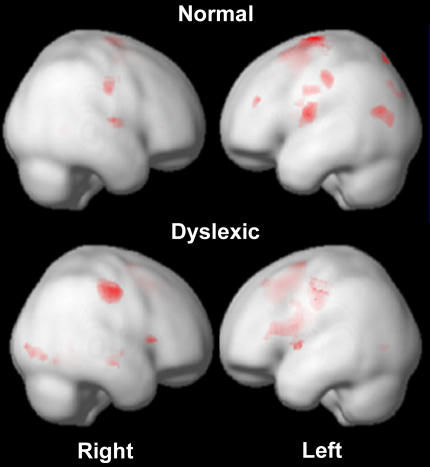
Different Activation Patterns in the Brains of Dyslexics As Compared to Normal Subjects in a Rhyming Task (Images courtesy of John Gabrieli, Stanford University.)

But the hypotheses that can be tested and the conclusions that can be drawn are still largely about group averages, not about the functionings of individual brains, and therein lies a second major caveat about the use of fMRI beyond the clinic. John Gabrieli, Associate Professor of Psychology at Stanford University, has shown that distinct activation patterns in the brains of dyslexic children normalize as they improve their reading skills (Figure 2). It seems like a small leap from there to including an fMRI as part of the workup for a schoolchild struggling in the classroom. But, Gabrieli cautions, that small leap in fact traverses a huge chasm, on one side of which is the group data from which conclusions are drawn in studies, and on the other side, the application of these conclusions to the individual child. “At the moment, fMRI would be among the most useless things to do. We would love to get it to the point that it would be useful [on an individual basis],” he says, but it's not there yet. “There is no single-subject reliability,” says Gabrieli. “Where we are now, I'm not aware of any applications for which it would be responsible to interpret an individual scan [based on group data].”

There are similar limitations to most other applications of fMRI—while conclusions can be made about aggregated data, individual scans are for the most part too hard to interpret. There is not yet any real understanding of how brain patterns change over time in an individual, or how interindividual differences should be interpreted in relation to the conclusions that are valid for groups. This makes fMRI an unlikely tool for job screening, for instance. While one study has shown a brain signature in a group of white people that is associated with racial bias, denying a particular individual a job on the basis of such a scan would likely lead straight to a lawsuit, with experts debating whether *this* scan in *this* individual on *this* day does or doesn't reflect his underlying racial attitudes.

On the other hand, Hirsch has used individual scans to help locate a patient's language structures that must be spared during neurosurgery. “If you are a neurosurgeon planning a resection, you don't want an average brain at all. Millimeters matter.” But her success is precisely because she is not using group data to make inferences about the individual—she is not leaping over the chasm, but instead is toiling entirely on the other side of it. “The goal is personalized medicine,” she says.

## A Little Guilty Knowledge Is a Dangerous Thing

Even this kind of personalized approach with fMRI is fraught with problems when researchers attempt to apply it outside the clinic, because of limitations in the technology itself. One researcher with firsthand knowledge of these problems is Daniel Langleben, Assistant Professor of Psychiatry at the University of Pennsylvania School of Medicine. In 2002, Langleben showed that when subjects were hiding information in an attempt to deceive (so-called guilty knowledge), they had intense activity in five distinct brain areas not seen when they were telling the truth. In effect, Langleben used the fMRI as a lie detector. It is potentially even more powerful than a standard polygraph test, he says, because there are thousands of brain regions which can be scanned for deception-triggered variation, versus only three variables—skin conductance, respiration, and blood pressure—used in the standard polygraph. Not surprisingly, Langleben got a lot of press after he announced his results, and his experiment led directly to speculation that we might eventually see fMRIs installed at airports, scanning the brains of would-be terrorists trying to deceive security screeners, or in courtrooms, catching perjurers red-handed (or perhaps red–anterior-cingulate-gyrused?).

Langleben is enthusiastic about the potential for an fMRI-based lie detector, and has even applied to the Department of Justice for a grant to develop the technology (they turned him down, saying it was too expensive). But he is also clear about how difficult it will be to get one that really works outside the highly structured confines of the research lab. “We are a long way from making a working polygraph,” he says. Even with a “Manhattan Project” type effort, he speculates it would take at least ten years. “There are still essential discoveries to make along the way,” he says, “and there's a good chance it would end in total failure.” It's not just a matter of developing the imaging technology, he stresses—“we'll need fundamental developments in semantics, too.” This is because “a lot still depends on how you ask the question”—the subtlest of differences can dramatically shift which areas of the brain respond. Given the sensitivity of the fMRI result to such seemingly minor perturbations, it's hard to imagine it could be reliably adapted to the hurly-burly of an airport security checkpoint.

Even well-performed scans done in topnotch clinics may not easily find their way into the courtroom. Perhaps the least likely use of fMRI is in determining if a defendant is telling the truth, according to Hank Greely, Professor of Law at Stanford Law School, since compelling someone on trial to submit to an fMRI could be seen as a violation of the Fifth Amendment right against self incrimination, just as giving spoken testimony against oneself is. On the other hand, says Greely, DNA samples and fingerprints can be compelled—whether a brain scan is more like testifying or more like submitting to a blood test is an open question. Still, for the moment, scanning under duress simply isn't feasible, since all you have to do to ruin a good scan is move your head. Motion-correcting algorithms can be used, but they are nowhere near advanced enough to correct for large-scale movements by an unwilling subject. It's much more likely that an fMRI of a willing defendant would be introduced to convince the jury he is telling the truth, or performed before trial to rule out an innocent suspect. While to Greely's knowledge fMRI evidence hasn't yet been used in court, “it's certain to be tried,” and the barrier to its admission will fall as both the reliability and the ease of administration increase. “The easier, the cheaper, the more pleasant a technique is, the more likely it is to be used in the legal system.”

Other forensic uses of fMRI are likely to arrive sooner rather than later. Could scans showing diminished impulse control—a function controlled by several regions of the brain, including the striatum and the ventromedial prefrontal cortex—be used to support more lenient sentencing, or even acquit a defendant, because he couldn't control his violent impulses? Or alternatively, will those same scans be used to argue for harsher sentences, since the defendant is clearly “hardwired” to commit similar crimes again? Courts already consider other factors, such as a history of child abuse, in an attempt to more fully understand the psychological state of the defendant. Will brain scans be seen as the ultimate “objective” look into the mind of the person on trial?

Deciding all these issues of admissibility will be judges who will need to weigh competing claims from lawyers with competing interests, backed up by expert witnesses with competing theories. Here, the desire to apply the science may rush ahead of its demonstrated validity.

Langleben, for one, doesn't think fMRI will be legitimately ready for the courtroom for a long time. On the other hand, he says, “if you want to abuse this technique and claim that it works, you can create tests that will produce results—I can see how it could be done. We know enough to rig it.” But still, he says, “we have all the tools we need to prevent this—there are enough people who are sufficiently honest [who would counter the premature use of fMRI in these contexts].”

For now, at least, given the problems inherent in current fMRI technology, the neuroethical nightmare scenarios of widespread brain scanning seem unlikely to come to pass, at least until radical advances make it far cheaper, much less invasive, far less sensitive to subtle perturbations, and with a much more robust ability to legitimately extrapolate from a finding about a group to a prediction about an individual. Where fMRI is concerned, “a penny for your thoughts” is currently more like “a million pennies for a group-averaged hemodynamic response to highly constrained stimuli under entirely artificial conditions.”

In light of this, bioethical concerns about fMRI applications should perhaps be viewed not as predictions of a certain future but rather as worstcase scenarios, a reminder of what we want to avoid. “It's a funny thing about the bioethics field,” says Greely. “The general approach is to look for bad news.”

While many of these “worst cases” seem highly unlikely to come to pass, Judy Illes, of the Stanford Center for Biomedical Ethics, thinks some action is warranted now, if only to generate a better understanding of the ethical dimensions of fMRI research. She notes that “bioethicists are often viewed as the ethics police,” but she doesn't see regulations as the right path to shape the future uses of fMRI. Instead, she thinks a coalition of involved parties—scientists, lawyers, ethicists, politicians—should work together to develop guidelines that all will find acceptable. “I'm not in the business of stopping anything.”

What everyone apparently already agrees on is the need for carefully designed experiments and cautious interpretation of the data. “A huge message in imaging is that you really have to look at the experimental setup at the common-sense level,” says Gabrieli, and avoid the tendency to “pick the most dramatic interpretation.” “The public needs to be reminded of the limitations of these findings,” agrees Hirsch. And as Langleben puts it, expressing his skepticism that there will ever be a one-size-fits-all, foolproof fMRI mind reader: “I don't think we'll ever be able to be stupid about it.”

## Abbreviation

fMRI: functional magnetic resonance imaging
